# Electronic Population-Based Depression Detection and Management Through Universal Screening in the Veterans Health Administration

**DOI:** 10.1001/jamanetworkopen.2022.1875

**Published:** 2022-03-10

**Authors:** Lucinda B. Leung, Karen Chu, Danielle Rose, Susan Stockdale, Edward P. Post, Kenneth B. Wells, Lisa V. Rubenstein

**Affiliations:** 1 Center for the Study of Healthcare Innovation, Implementation, and Policy, VA Greater Los Angeles Healthcare System, Los Angeles, California; 2 Division of General Internal Medicine and Health Services Research, UCLA David Geffen School of Medicine, Los Angeles, California; 3 VA Ann Arbor, Center for Clinical Management Research, Ann Arbor, Michigan; 4 Department of Medicine, University of Michigan Medical School, Ann Arbor; 5 Department of Psychiatry and Biobehavioral Sciences, UCLA Semel Institute for Neuroscience and Human Behavior, Los Angeles, California; 6 RAND Corporation, Santa Monica, California

## Abstract

**Importance:**

In 2016, the US Preventive Services Task Force newly recommended universal screening for depression, with the expectation that screening would be associated with appropriate treatment. Few studies have been able to assess the population-based trajectory from screening to receipt of follow-up and treatment for individuals with depression.

**Objective:**

To examine adherence to guidelines for follow-up and treatment among primary care patients who newly screened positive for depression in the Veterans Health Administration (VA).

**Design, Setting, and Participants:**

This retrospective cohort study used VA electronic data to identify patients who newly screened positive for depression on the 2-item Patient Health Questionnaire at 82 primary care VA clinics in California, Arizona, and New Mexico between October 1, 2015, and September 30, 2019. Data analysis was performed from December 2020 to August 2021.

**Main Outcomes and Measures:**

Receipt of guideline-concordant care for screen-positive patients who were determined by clinicians as having depression was assessed. Timely follow-up (within 84 days of screening) was defined as receiving 3 or more mental health specialty visits, 3 or more psychotherapy visits, or 3 or more primary care visits with a depression diagnosis according to the *International Statistical Classification of Diseases and Related Health Problems, Tenth Revision*. Completing at least minimal treatment (within 12 months) was defined as having 60 days or more of antidepressant prescriptions filled, 4 or more mental health specialty visits, or 3 or more psychotherapy visits.

**Results:**

The final cohort included 607 730 veterans (mean [SD] age, 59.4 [18.2] years; 546 516 men [89.9%]; 339 811 non-Hispanic White [55.9%]); 8%, or 82 998 of 997 185 person-years, newly screened positive for depression. Clinicians identified fewer than half with depression (15 155 patients), of whom 32% (5034 of 15 650 person-years) met treatment guidelines for timely follow-up and 77% (12 026 of 15 650 person-years) completed at least minimal treatment. Younger age (odds ratio, 0.990; 95% CI, 0.986-0.993; *P* < .001), Black race (odds ratio, 1.19; 95% CI, CI 1.05-1.34; *P* = .01), and having comorbid psychiatric diagnoses were significantly associated with timely follow-up. Individual quality metric components (eg, medication or psychotherapy) were associated differently with overall quality results among patient groups, except for age.

**Conclusions and Relevance:**

In this cohort study, most patients met the guidelines for completing at least minimal treatment, but only a minority received timely follow-up after screening positive and being identified as having depression. More research is needed to understand whether the discrepancy between patients who screened positive and patients identified as having depression reflects a gap in recognition of needed care.

## Key Points

**Question:**

Among health care systems that implement universal depression screening, do patients who screen positive for depression receive timely follow-up and treatment?

**Findings:**

In a cohort study of 607 730 patients at 82 Veterans Health Administration primary care clinics from 2015 to 2019, 15 155 patients newly screened positive on the 2-item Patient Health Questionnaire and also were detected by clinicians as having depression; 77% met guidelines for completing at least minimal treatment in 1 year, but only 32% received clinical follow-up within 3 months of screening.

**Meaning:**

These findings suggest that screening paired with integrated services offers a reasonable opportunity to engage primary care patients in depression treatment, supporting collaborative care’s extensive evidence base; yet, access to timely services remains limited.

## Teaser

This cohort study examines adherence to guidelines for follow-up and treatment among primary care patients who newly screened positive for depression in the Veterans Health Administration (VA).

## Introduction

Major depressive disorder is the leading cause of disability worldwide.^1^ In 2016, the US Preventive Services Task Force (USPSTF) newly recommended universal depression screening in the general adult population, with the expectation that screening would be linked to appropriate treatment.^2^ USPSTF recognized that staff-assisted depression care directed at ensuring accurate diagnosis, appropriate follow-up, and effective treatment conferred substantial improvement in clinical outcomes and was increasingly available in primary care settings. Yet, others have disagreed^3^ and cited that evidence supporting the benefits of universal depression screening is too limited. For example,^4^ 2013 guidelines from the Canadian Task Force on Preventive Health Care recommended against routine depression screening to avoid depression overdiagnosis. Knowledge gaps in depression screening remain because few health care systems have been able to pragmatically study the population-based trajectory from screening to follow-up and treatment for those who have depression.

For more than 2 decades, the Veterans Health Administration (VA) has mandated annual depression screening in primary care^5^ and currently achieves nearly universal screening rates among clinics nationally.^6^ Primary care nurses conduct initial screening; then, primary care clinicians follow up with patients who screen positive for confirmation and treatment of depression. Primary care mental health integration teams also provide diagnostic support and collaboratively treat mental and behavioral health conditions, with a focus on mild-to-moderate severity depression and anxiety.^7^ Primary care mental health integration teams (eg, psychologists, psychiatrists, and nurse care managers) are readily available in person or virtually throughout primary care clinics nationally, in addition to more intensive services provided through traditional mental health specialty referrals. The VA system is thereby equipped to address patients who are identified as having mental health needs, especially those with clinical depression.

In the US, there is an increasing call for depression to be addressed at the population level and for its detection and management to be done through public health approaches.^8^ Other health care systems similarly embarking on guideline-concordant depression care (via screening, follow-up, and treatment) may benefit from anticipatory guidance on the volume of positive screens and treatment seekers within a primary care population. We use a new method to generate electronic population-based depression care quality measures to add to the limited evidence base on patient outcomes from universal depression screening. This cohort study aims to examine adherence to guidelines for follow-up and treatment among primary care patients who newly screened positive for depression in the VA.

## Methods

### Study Design and Cohort

The VA Greater Los Angeles institutional review board approved this study. Because the evaluation efforts were part of an ongoing quality improvement effort at the VA, the institutional review board deemed this study to be nonhuman participants research and, therefore, exempt from informed consent requirements. This study followed the Strengthening the Reporting of Observational Studies in Epidemiology (STROBE) reporting guideline.

In a retrospective cohort study, we identified primary care patients among 82 clinics in 1 VA region between October 1, 2015, and September 30, 2019, using VA electronic data. Patient data were drawn from 11 hospital-based and 71 community-based VA clinics in Southern California, Arizona, and New Mexico that administered depression screening via the 2-item Patient Health Questionnaire.

**Figure 1.**
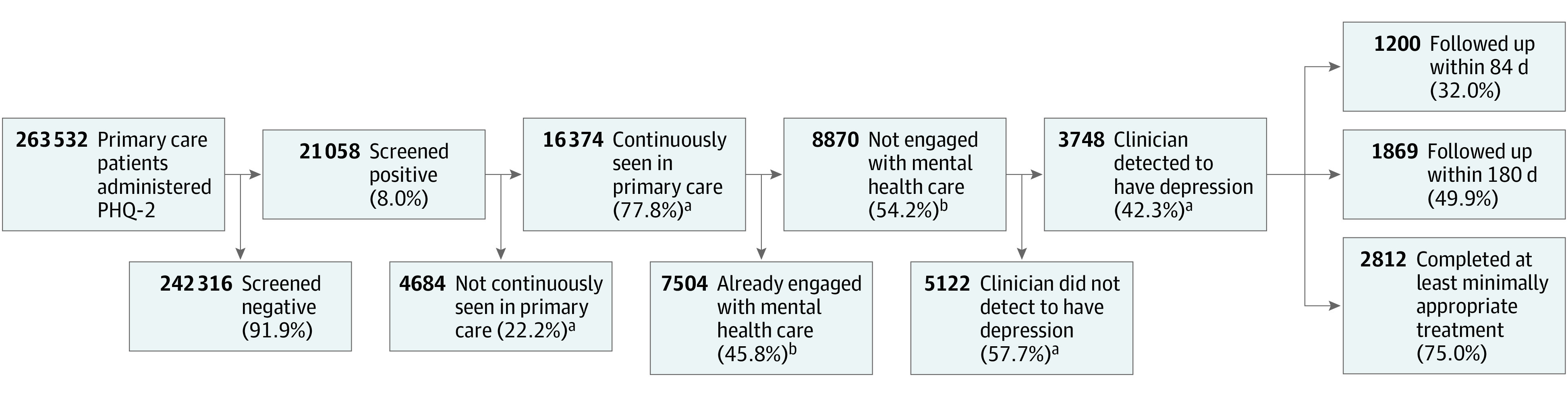
Outcomes Among Primary Care Patients Screened for Depression in Fiscal Year 2019 Flowchart shows sample sizes and corresponding rates of depression screening and management in our study population for the most recent fiscal year. Sample sizes and rates shown in the text differ and reflect observations aggregated from all 4 study years. ^a^Refers to the 1-year prospective time frame when numbers were obtained. ^b^Refers to the 6-month retrospective time frame when numbers were obtained.

This study expanded on recognized depression care quality metrics^9^ by newly incorporating depression screening data using administrative and pharmacy databases from VA’s Corporate Data Warehouse. First, we identified 71 421 patients who screened positive for depression in primary care clinics by scoring 3 or higher on the 2-item Patient Health Questionnaire. Second, we restricted the cohort to patients who visited the same primary care site at least 1 additional time, allowing opportunity for clinical intervention. Third, to isolate patients with a new episode of depression, we further restricted the cohort to those who had not already received a diagnosis of depression (ie, *International Statistical Classification of Diseases and Related Health Problems, Tenth Revision [ICD-10]* depression diagnosis) or engaged in mental health care (ie, ≥60 days of antidepressant prescriptions, ≥4 mental health specialty visits, or ≥3 psychotherapy visits) (eTable 1, eTable 2, eTable 3, and eTable 4 in the Supplement) within the past 6 months. Among our cohort who screened positive and met the aforementioned criteria, we further identified patients who were also detected by a clinician as having depression via diagnostic coding or antidepressant prescription within 12 months of screening positive (Figure 1 shows data for fiscal year 2019).

### Dependent Variables 

Our 3 study outcomes were population-based depression care quality metrics established and detailed in prior research.^9^ Measures were constructed on the basis of VA and National Committee for Quality Assurance guidelines and then were agreed on by a modified Delphi panel of VA and non-VA experts. We used 2 measures of timely follow-up, defined as 3 or more mental health specialty visits, 3 or more psychotherapy visits, or 3 or more primary care visits with a depression *ICD-10* diagnosis within (1) 84 days and (2) 180 days. Also, we measured completion of at least minimally appropriate treatment (ie, having ≥60 days of antidepressant prescriptions, ≥4 mental health specialty visits, or ≥3 psychotherapy visits within 12 months of screening positive). Prescriptions with subtherapeutic doses and with nondepression indications (or keywords written on the dosing instructions) were excluded as antidepressant medications. We dichotomized outcomes as patients either having received or not received guideline-concordant depression care in each study year.

### Independent Variables

This study comprehensively examined available patient and clinic characteristics known or hypothesized to be associated with depression care quality. Data sources included VA’s Corporate Data Warehouse, National Patient Care Database, Vital Status File, and Site Tracking System. Patient covariates consisted of age, sex, self-reported race and ethnicity (ie, non-Hispanic White, non-Hispanic Black, Hispanic, other [American Indian or Alaska Native, Asian, and Native Hawaiian or other Pacific Islander], or unknown or missing), marital status, and income proxies (because patients may be eligible for VA care on the basis of a service-connected disability or exempt from copayment on the basis of having low financial means). Race and ethnicity were assessed in this study because disparities in mental health treatment by racial and ethnic group have been reported.^10^ We used *ICD-10* codes in outpatient and inpatient visits to identify mental health diagnoses (ie, depression, anxiety, posttraumatic stress disorder [PTSD], alcohol and substance use disorders, and serious mental illness [specifically, schizophrenia and bipolar disorder]) (eTable 5 in the Supplement) and to calculate Charlson Comorbidity Index scores to risk-adjust physical health for each patient during each study year. Finally, we adjusted for whether patients’ assigned clinics were community-based vs hospital-based, rural vs urban location, and 5000 or more vs fewer assigned primary care patients (as a proxy for clinic size).

### Statistical Analysis

For descriptive purposes, we calculated unadjusted, aggregate rates of achieving depression care quality measures across our cohort and then examined patient and clinic characteristics for (1) screen-positive patients and (2) screen-positive patients who were also detected by clinicians as having depression, using *t* and χ^2^ tests. We analyzed patient and clinic characteristics among those who met and did not meet depression care quality outcome measures, using χ^2^ tests.

This study used multilevel regression models to examine for associations between depression care quality and the aforementioned patient and clinic characteristics among all study patients. We included year and health care system fixed effects to account for secular trends and invariant organizational characteristics. Patient random effects were included to account for the possibility of patients having multiple nonindependent observations during the 4 study years. SEs were also adjusted to account for clustering of patients within clinics. Given the dichotomous distributions of our quality outcomes, we reported odds ratios (ORs) and 95% CIs from multilevel logistic regressions in adjusted models. Estimates were also presented as probabilities (and SEs were calculated via the delta method), with all covariates held constant at their means. In additional analyses, we examined the contribution of each quality metric component (eg, counts of antidepressant prescriptions and psychotherapy visits) and reported incidence rate ratios (IRRs) and 95% CIs from multilevel negative binomial regression models. Finally, sensitivity analyses were conducted to examine for interactions between patient demographic characteristics (eg, age, sex, race, and ethnicity) and between demographic and clinic variables (eg, rurality, hospital-based vs community-based), to account for known demographic differences among veterans across age groups and clinic locations. For all models, we determined significance by using a 2-tailed α = .05. Data were analyzed in Stata statistical software version 15.1 (StataCorp). Data analysis was performed from December 2020 to August 2021.

## Results

Our study included 607 730 veterans (mean [SD] age, 59.4 [18.2] years; 546 516 men [89.9%]; 339 811 non-Hispanic White [55.9%]). Approximately 8% (82 998 of 997 185 person-years, or 71 421 patients) screened positive for new depressive symptoms in VA primary care. Among those who screened positive, 80% (66 305 of 82 998 person-years, or 57 779 patients) continued to receive primary care services that year in the same site, allowing opportunities for clinical intervention. Among those, 56% (37 063 of 66 305 person-years) had not already engaged in mental health care (within the past 6 months). We then examined the remaining 33 694 patients who newly screened positive for depression in our study cohort and noted several patient (ie, age, race, ethnicity, and comorbid conditions) and clinic characteristics to be significantly associated with depression care quality (Table 1 and eTable 6 in the Supplement).

**Table 1.** Characteristics of Screen-Positive Patients and Patients Also Detected by Clinicians as Having Depression

Characteristic	Patients, No. (%)^a^				
	Total screen positive (n = 33 694)	Screen positive and detected by clinician to have depression (n = 15 155)			
		Total	Follow-up within 84 d	Follow-up within 180 d	Minimally appropriate treatment
Age, mean (SD), y	56.31 (17.33)	51.56 (17.00)	47.95 (16.05)	48.66 (16.30)	50.38 (16.71)
Sex					
Male	30 577 (91)	13 308 (88)	4266 (87)	6688 (87)	10 208 (87)
Female	3117 (9)	1847 (12)	658 (13)	988 (13)	1447 (12)
Race and ethnicity					
Black	4677 (14)	2340 (15)	871 (18)	1311 (17)	1818 (16)
Hispanic	6785 (20)	3277 (22)	1083 (22)	1698 (22)	2557 (22)
White	16 900 (50)	7090 (47)	2176 (44)	3421 (45)	5435 (47)
Other^b^	1422 (4)	713 (5)	221 (4)	360 (5)	543 (5)
Unknown or missing	3910 (12)	1735 (11)	573 (12)	886 (12)	1332 (11)
Marital status					
Married	14 194 (42)	6053 (40)	1785 (36)	2874 (37)	4703 (40)
Single or previously married	19 500 (58)	9102 (60)	3139 (64)	4802 (63)	6982 (60)
Means test					
Exempt	3381 (10)	3550 (23)	1246 (25)	1836 (24)	2664 (23)
Nonexempt	3381 (10)	1521 (10)	481 (10)	783 (10)	1160 (10)
Any copayment required	3735 (11)	1572 (10)	535 (11)	834 (11)	1202 (10)
Missing	18 088 (54)	8512 (56)	2662 (54)	4223 (55)	6659 (57)
Service-connected disability					
0%	892 (3)	384 (3)	123 (3)	184 (2)	288 (2)
1%-50%	6936 (21)	3180 (21)	1087 (22)	1668 (22)	2464 (21)
51%-100%	13 238 (39)	6254 (41)	1776 (36)	2969 (39)	4918 (42)
Missing	12 628 (37)	5337 (35)	1938 (39)	2855 (37)	4015 (34)
Charlson Comorbidity Index score					
0	17912 (53)	9049 (57)	2350 (66)	4908 (64)	7156 (61)
1	7115 (21)	2949 (19)	893 (18)	1455 (19)	2249 (19)
≥2	8667 (26)	3157 (21)	781 (16)	1313 (17)	2280 (20)
Mental health or substance use disorder					
Anxiety disorder	8162 (24)	5807 (38)	2562 (52)	3750 (49)	4838 (41)
Posttraumatic stress disorder	11 330 (34)	7109 (47)	2772 (56)	4321 (56)	6006 (51)
Serious mental illness	1199 (4)	697 (5)	348 (7)	507 (7)	613 (5)
Alcohol use disorder	3304 (10)	2056 (14)	974 (20)	1369 (18)	1730 (15)
Substance use disorder	1646 (5)	1075 (7)	564 (11)	765 (10)	927 (8)
Clinic characteristics					
Rural	2611 (8)	911 (6)	184	300	649
Small	8948 (27)	3592 (24)	910	1615	2781
Community based	21 468 (64)	9618 (63)	13 231	12 224	10 877

^a^ Numbers represent unique patients. If patients were counted in multiple fiscal years, we reported characteristics associated with the most recent fiscal year (eg, latest age).

^b^ Other includes American Indian or Alaska Native, Asian, and Native Hawaiian or other Pacific Islander.

**Figure 2.**
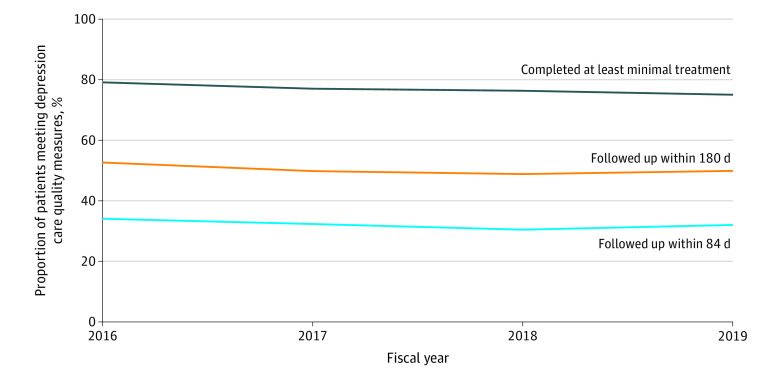
Depression Follow-up and Treatment Rates Among Newly Diagnosed Primary Care Patients for Fiscal Years 2016 to 2019

However, fewer than one-half of screen-positive patients were detected by clinicians as having depression via diagnosis and/or antidepressant prescription (15 155 patients). When clinicians detected depression among patients who screened positive, 32% (5034 of 15 650 person-years) met treatment guidelines for appropriate timely follow-up by receiving 3 or more mental health specialty visits, 3 or more psychotherapy visits, or 3 or more primary care visits with a depression *ICD-10* diagnosis within 84 days of screening; 77% (12 026 of 15 650 person-years) completed at least minimally appropriate treatment by having 60 days or more of antidepressant prescriptions filled, 4 or more mental health specialty visits, or 3 or more psychotherapy visits within 12 months of screening. Figure 1 shows data for fiscal year 2019. Percentages were stable across all 4 study years at the VA (Figure 2).

Certain patient characteristics remained significantly associated with depression care quality among screen-positive patients who were also detected by clinicians as having depression (Table 2). Younger age (OR for receiving treatment, 0.990; 95% CI, 0.986-0.993; *P* < .001) and comorbid mental illness were factors significantly associated with higher depression care quality. Probabilities for timely follow-up were 33% among patients younger than 45 years and 21% among patients older than 75 years; probabilities for receiving treatment were 81% among the younger patients and 71% among the older patients. Mental health comorbidities were important factors associated with high-quality depression care; as an example, probabilities for timely follow-up were 38% among patients with PTSD and 24% for patients without PTSD, probabilities for receiving treatment were 85% among those with PTSD and 72% among those without PTSD. Although having physical health comorbidities was not associated with receipt of treatment, it was an important factor associated with not receiving timely follow-up (OR, 0.80; 95% CI, 0.70-0.90; *P* < .001), with probabilities of 12% for Charlson Comorbidity Index scores of 2 or higher vs 14% for Charlson Comorbidity Index scores of 0. Black race was significantly associated with higher odds of timely follow-up vs White race (OR, 1.19; 95% CI, 1.05-1.34; *P* = .01), but race was not otherwise associated with treatment completion. When controlling for all covariates at their means, probabilities for follow-up among Black and White patients were 32% and 29%, respectively.

**Table 2.** Associations Between Receipt of Timely Depression Follow-up and Treatment and Various Patient Characteristics Among All Screen-Positive Patients Who Were Also Detected by Clinicians as Having Depression^a^

Characteristic	Follow-up within 84 d		Follow-up within 180 d		Minimally appropriate treatment	
	OR (95% CI)	*P* value	OR (95% CI)	*P* value	OR (95% CI)	*P* value
Age	0.990 (0.986-0.993)	<.001	0.987 (0.98-0.99)	<.001	0.984 (0.98-0.99)	<.001
Sex						
Male	1 [Reference]	NA	1 [Reference]	NA	1 [Reference]	NA
Female	1.02 (0.90-1.16)	.76	0.96 (0.85-1.08)	.53	1.11 (0.93-1.33)	.25
Race and ethnicity						
Black	1.19 (1.05-1.34)	.01	1.22 (1.08-1.37)	<.001	1.03 (0.87-1.22)	.73
Hispanic	1.02 (0.91-1.13)	.78	1.02 (0.92-1.13)	.68	0.93 (0.83-1.08)	.35
White	1 [Reference]	NA	1 [Reference]	NA	1 [Reference]	NA
Other^b^	0.98 (0.80-1.20)	.86	1.06 (0.88-1.28)	.52	0.81 (0.62-1.05)	.12
Unknown or missing	1.16 (1.01-1.33)	.03	1.15 (1.02-1.31)	.03	1.02 (0.85-1.22)	.86
Marital status						
Married	1 [Reference]	NA	1 [Reference]	NA	1 [Reference]	NA
Single or previously married	0.1 (0.91-1.09)	.91	0.99 (0.91-1.08)	.83	1.20 (1.06-1.36)	<.001
Means test						
Exempt	1 [Reference]	NA	1 [Reference]	NA	1 [Reference]	NA
Nonexempt	0.87 (0.74-1.07)	.21	1.05 (0.88-1.25)	.58	0.97 (0.76-1.24)	.81
Any copayment required	0.92 (0.79-1.07)	.28	1.03 (0.89-1.19)	.72	0.99 (0.81-1.21)	.92
Missing	0.98 (0.86-1.11)	.71	0.98 (0.88-1.11)	.79	0.99 (0.85-1.18)	.99
Service-connected disability						
0%	1 [Reference]	NA	1 [Reference]	NA	1 [Reference]	NA
1%-50%	1.03 (0.77-1.37)	.86	1.07 (0.82-1.40)	.62	1.07 (0.74-1.54)	.73
51%-100%	0.67 (0.50-0.89)	.01	0.76 (0.58-9.88)	.04	0.95 (0.66-1.35)	.77
Missing	1.22 (0.93-1.60)	.15	1.28 (0.10-1.65)	.05	1.04 (0.74-1.46)	.83
Charlson Comorbidity Index score						
0	1 [Reference]	NA	1 [Reference]	NA	1 [Reference]	NA
1	0.93 (0.83-1.05)	.23	1.08 (0.97-1.20)	.15	1.13 (0.97-1.31)	.12
≥2	0.8 (0.70-0.90)	<.001	0.91 (0.82-1.02)	.10	1.03 (0.89-1.20)	.68
Mental health or substance use disorder						
Anxiety disorder	2.38 (2.08-2.71)	<.001	2.65 (2.31-3.04)	<.001	2.16 (1.79-2.60)	<.001
Posttraumatic stress disorder	2.04 (1.82-2.31)	<.001	2.63 (2.29-3.02)	<.001	2.92 (2.29-3.72)	<.001
Serious mental illness	2.08 (1.69-2.56)	<.001	2.79 (2.21-3.53)	<.001	2.69 (1.86-3.90)	<.001
Alcohol use disorder	1.70 (1.46-1.90)	<.001	1.65 (1.44-3.53)	<.001	1.46 (1.20-1.77)	<.001
Substance use disorder	1.77 (1.49-2.10)	<.001	1.77 (1.48-2.11)	<.001	1.82 (1.38-2.41)	<.001

Abbreviations: NA, not applicable; OR, odds ratio.

^a^ There were a total of 15 650 person-years.

^b^ Other includes American Indian or Alaska Native, Asian, and Native Hawaiian or other Pacific Islander.

Each quality metric component (eg, antidepressant treatment, psychotherapy visits), appeared to contribute differently among patient groups, apart from age. Among patients with and without mental health comorbidities, results were similar across quality metric components, except PTSD. Among patients with and without PTSD, we found that mental health specialty (IRR, 1.18; 95% CI, 1.10-1.27; *P* < .001) and psychotherapy visits (IRR, 1.63; 95% CI, 1.52-1.75; *P* < .001) were associated with higher rates of timely follow-up; there was no difference in primary care visits. Similarly, among patients with Charlson Comorbidity Index score of 2 or higher vs those with a score of 0, we observed lower rates of timely follow-up through mental health specialty (IRR, 0.89; 95% CI, 0.81-0.99; *P* = .04) and psychotherapy visits (IRR, 0.86; 95% CI, 0.77-0.97; *P* = .01); there was no difference in primary care visits. For Black veterans compared with White veterans, greater use of mental health specialty care (IRR, 1.12; 95% CI, 1.02-1.23; *P* = .01) was associated with differences in timely follow-up. Although we did not see racial or ethnic differences in overall rates of treatment completion, we saw differences in use of different treatment types between White veterans and those from minority groups. Black veterans (IRR, 0.8; 95% CI, 0.76-0.86; *P* < .001) and Hispanic veterans (IRR, 0.88; 95% CI, 0.76-0.93; *P* < .001) had lower rates of treatment with antidepressant medication but had higher rates of mental health specialty visits (Black veterans, IRR, 1.18; 95% CI, 1.1-1.27; *P* < .001) and psychotherapy visits (Hispanic veterans, IRR, 1.11; 95% CI, 1.02-1.20; *P* = .01), compared with White veterans. Although no sex differences were observed among quality metrics, we noted that of individual metric components, primary care visits with a depression *ICD-10* diagnosis were significantly more likely for women (IRR, 1.82; 95% CI, 1.44-2.31; *P* < .001) than men. Finally, no significant interactions were identified among tested patient demographic and clinic variables in sensitivity analyses.

## Discussion

The VA has invested heavily in screening as an important part of the pathway for patients to initiate and access mental health treatment,^11^ allowing us to assess results of a primary care population receiving USPSTF guideline-concordant depression care.^2^ For some researchers, without more data, routine use of depression screening in medical settings remains controversial.^3,4^ In the absence of randomized clinical trials, our large observational cohort study attempts to fill knowledge gaps surrounding systemwide implementation of depression screening. We found that 8% of patients screened positive among primary care populations in our integrated VA health care system, but clinicians identified fewer than one-half of screen-positive patients as having depression. Those who were not detected as having depression are likely an unknown mix of patients who were appropriately assessed and were found to not meet the criteria for a depression diagnosis (desired care process) and patients who were never appropriately assessed or assessed with no diagnosis recorded (undesired care process). More research is needed to understand whether this indicates a gap in recognition of needed care or overdetection from universal screening in the VA.

Although most patients with depression met guidelines for completing treatment within a year of screening positive, only a minority received timely clinical follow-up within 3 to 6 months. Trends did not improve over time for the approximately two-thirds and one-half of screen-positive patients who were detected by clinicians as having depression and did not receive follow-up visits within 3 to 6 months. Across all years, approximately one-quarter of these patients did not receive at least minimal treatment within 1 year. As a system that aims to lead in care of PTSD (a common comorbidity to depression)^12^ and to prioritize suicide prevention (for which depression is a major risk factor),^13^ improving timeliness of follow-up and treatment after a positive depression screen remains necessary in the VA. Similarly, other health care systems have struggled and cited that only 36% of patients with newly diagnosed depression even start medication and/or complete 1 psychotherapy visit during the first 3 months.^14^ Nonetheless, screening paired with accessible mental health services generally offered reasonable opportunity to engage VA primary care patients in treatment, supporting the extensive evidence base for collaborative care of depression.^15^

This study also highlights notable differences in depression care quality between patient groups. We found that timely follow-up and treatment continued to lag for geriatric patients^16^ and those with chronic physical health comorbidities,^17^ likely related to competing demands such as specialty care. Patients who had comorbid mental health conditions, however, fared well in the receipt of high-quality depression care, as noted before,^18^ likely a testament to VA-specific services for those with PTSD or serious mental illness (eg, Mental Health Intensive Case Management).^19^ In a male-dominated health system, we again noted sex differences in mental health care access, seemingly occurring preferentially for women through primary care services.^20^ Although some disparities between patient groups have been remedied by an integrated health system that prioritizes mental health care accessibility, efforts to improve the timeliness of care after a positive depression screen and treatment of geriatric patients and patients with physical health comorbidities remain necessary.

A new and noteworthy observation pertains to the absence of and possible reversal of oft-seen racial disparities in overall mental health care access across health care systems. Although it was again noted that Black veterans were treated with antidepressant medication at lower rates than White veterans,^10^ we did not see racial disparities in overall rates of treatment completion when we fully accounted for veterans who chose to seek nonmedication treatment through mental health specialty care. The VA has striven for increasing accessibility of psychotherapy as part of integrated primary care teams,^7^ which is the treatment modality that is preferred by patients, especially those in racial and ethnic minority groups.^21^ Such systemwide changes may have resulted in previously documented racial and ethnic disparities in guideline-concordant depression treatment^10^ to seemingly be eliminated in the VA. Depression is often underdetected and undertreated among minoritized groups in the US.^22^ This study continues to support that systematic quality improvement of screening and treatment are potential tools to mitigate racial and ethnic disparities in mental health care.^23^

### Limitations

To our knowledge, this study is one of the first to examine timely follow-up and treatment for primary care patients who screen positive for depression in an integrated health care system, but several limitations are worth noting. First, because we leveraged existing electronic databases, we were unable to investigate patient-reported quality outcomes, such as depression symptoms or quality of life. Second, our study excluded fewer than 20% of patients who did not return for primary care after screening. Our study cohort was designed to assess care quality for patients who had the opportunity to receive continuity primary care. Focusing on patients who drop out of VA care, often because they have had an acute problem when traveling, are switching VA clinics, or are seeking non-VA care, requires a different study approach and lead to different implications than were the case for this study. Third, similar to other studies based on administrative data, coding inaccuracies (eg, inactive depression diagnoses being incorrectly recoded, possible discrepancies related to antidepressant prescriptions without listed indications) may be limitations. Fourth, although the results may not be generalizable beyond our study population of mostly male veterans in 1 VA region, prior studies show that the large majority of mental health services are delivered to veterans by the VA.^24^

## Conclusions

With increasing recognition of population-level impacts of mental health disorders, an epidemiological framework for mapping access to care for patients who screen positive for depression in VA primary care is methodologically crucial. In the VA, we observed that universal screening yields a stable proportion of patients who screen positive for depression, of whom the minority receive timely follow-up but the majority receive guideline-concordant treatment. Although the VA’s investments in depression screening and subsequent follow-up care and treatment have seemingly closed some disparity gaps (in mental health comorbidities and race), several others remain (in age and in physical health comorbidities). Continued research in mapping access to care pathways for patients who screen positive for depression is needed to ultimately improve upon patient health outcomes for VA and other health care systems.

## Notes

Supplement. **eTable 1.** *ICD-10 *Codes Used for Depression Diagnosis **eTable 2.** List of Drugs Classified as Antidepressant Medication in Study **eTable 3.*** Current Procedural Terminology (CPT)* Codes for Psychotherapy **eTable 4.** VA Clinic Stop Codes for Mental Health Specialty Visits **eTable 5.** *ICD-10* Codes Used for Other Mental Health Diagnoses in Study **eTable 6. **Associations Between Receipt of Timely Depression Follow-up and Treatment and Various Patient Characteristics (Among All Screen-Positive Patients)
