# Supplementary material for: Electronic Population-Based Depression Detection and Management Through Universal Screening in the Veterans Health Administration
Source: JAMA Netw Open. 2022 Mar 10;5(3):e221875. doi: 10.1001/jamanetworkopen.2022.1875 (PMC8914576; doi:10.1001/jamanetworkopen.2022.1875)
Supplement: Supplement. — eTable 1. ICD-10 Codes Used for Depression Diagnosis eTable 2. List of Drugs Classified as Antidepressant Medication in Study eTable 3. Current Procedural Terminology (CPT) Codes for Psychotherapy eTable 4. VA Clinic Stop Codes for Mental Health Specialty Visits eTable 5. ICD-10 Codes Used for Other Mental Health Diagnoses in Study eTable 6. Associations Between Receipt of Timely Depression Follow-up and Treatment and Various Patient Characteristics (Among All Screen-Positive Patients) [file jamanetwopen-e221875-s001.pdf]

Supplemental Online Content

Leung LB, Chu K, Rose D, et al. Electronic population-based depression detection and management through universal screening in the Veterans Health Administration. *JAMA Netw Open.* 2022;5(3):e221875. doi:10.1001/jamanetworkopen.2022.1875

eTable 1. *ICD-10* Codes Used for Depression Diagnosis
eTable 2. List of Drugs Classified as Antidepressant Medication in Study
eTable 3. *Current Procedural Terminology (CPT)* Codes for Psychotherapy
eTable 4. VA Clinic Stop Codes for Mental Health Specialty Visits
eTable 5. *ICD-10* Codes Used for Other Mental Health Diagnoses in Study
eTable 6. Associations Between Receipt of Timely Depression Follow-up and Treatment and Various Patient Characteristics (Among All Screen-Positive Patients)

This supplemental material has been provided by the authors to give readers additional information about their work.

© 2022 Leung LB et al. *JAMA Network Open.*

eTable 1. *ICD-10* Codes Used for Depression Diagnosis

***ICD-10* code**	***ICD-10* Description**
F32.0	MAJOR DEPRESSIVE DISORDER, SINGLE EPISODE, MILD
F32.1	MAJOR DEPRESSIVE DISORDER, SINGLE EPISODE, MODERATE
F32.2	MAJOR DEPRESSIVE DISORDER, SINGLE EPISODE, SEVERE WITHOUT PSYCHOTIC FEATURES
F32.3	MAJOR DEPRESSIVE DISORDER, SINGLE EPISODE, SEVERE WITH PSYCHOTIC FEATURES
F32.4	MAJOR DEPRESSIVE DISORDER, SINGLE EPISODE, IN PARTIAL REMISSION
F32.5	MAJOR DEPRESSIVE DISORDER, SINGLE EPISODE, IN FULL REMISSION
F32.8	OTHER DEPRESSIVE EPISODES
F32.89	OTHER SPECIFIED DEPRESSIVE EPISODES
F32.9	MAJOR DEPRESSIVE DISORDER, SINGLE EPISODE, UNSPECIFIED
F33.0	MAJOR DEPRESSIVE DISORDER, RECURRENT, MILD
F33.1	MAJOR DEPRESSIVE DISORDER, RECURRENT, MODERATE
F33.2	MAJOR DEPRESSIVE DISORDER, RECURRENT SEVERE WITHOUT PSYCHOTIC FEATURES
F33.3	MAJOR DEPRESSIVE DISORDER, RECURRENT, SEVERE WITH PSYCHOTIC SYMPTOMS
F33.40	MAJOR DEPRESSIVE DISORDER, RECURRENT, IN REMISSION, UNSPECIFIED
F33.41	MAJOR DEPRESSIVE DISORDER, RECURRENT, IN PARTIAL REMISSION
F33.42	MAJOR DEPRESSIVE DISORDER, RECURRENT, IN FULL REMISSION
F33.8	OTHER RECURRENT DEPRESSIVE DISORDERS
F33.9	MAJOR DEPRESSIVE DISORDER, RECURRENT, UNSPECIFIED
F06.30	MOOD DISORDER DUE TO KNOWN PHYSIOLOGICAL CONDITION, UNSPECIFIED
F39.	UNSPECIFIED MOOD [AFFECTIVE] DISORDER
F34.1	DYSTHYMIC DISORDER
F43.21	ADJUSTMENT DISORDER WITH DEPRESSED MOOD
F31.30	BIPOLAR DISORDER, CURRENT EPISODE DEPRESSED, MILD OR MODERATE SEVERITY, UNSPECIFIED
F31.31	BIPOLAR DISORDER, CURRENT EPISODE DEPRESSED, MILD
F31.32	BIPOLAR DISORDER, CURRENT EPISODE DEPRESSED, MODERATE
F31.4	BIPOLAR DISORDER, CURRENT EPISODE DEPRESSED, SEVERE, WITHOUT PSYCHOTIC FEATURES
F31.5	BIPOLAR DISORDER, CURRENT EPISODE DEPRESSED, SEVERE, WITH PSYCHOTIC FEATURES
F31.75	BIPOLAR DISORDER, IN PARTIAL REMISSION, MOST RECENT EPISODE DEPRESSED
F31.76	BIPOLAR DISORDER, IN FULL REMISSION, MOST RECENT EPISODE DEPRESSED

© 2022 Leung LB et al. *JAMA Network Open.*

eTable 2. List of Drugs Classified as Antidepressant Medication in Study

DrugClass	DrugClassification
CN600	ANTIDEPRESSANTS
CN601	TRICYCLIC ANTIDEPRESSANTS
CN602	MONAMINE OXIDASE INHIBITOR ANTIDEPRESSANTS
CN609	ANTIDEPRESSANTS, OTHER
	**DrugName**
	AMITRIPTYLINE
	AMOXAPINE
	BUPROPION
	CITALOPRAM
	CLOMIPRAMINE
	DESIPRAMINE
	DESVENLAFAXINE
	DOXEPIN
	DULOXETINE
	ESCITALOPRAM
	FLUOXETINE
	FLUVOXAMINE
	IMIPRAMINE
	ISOCARBOXAZID
	LEVOMILNACIPRAN
	MAPROTILINE
	MILNACIPRAN
	MIRTAZAPINE
	NEFAZODONE
	NOMIFENSINE
	NORTRIPTYLINE
	PAROXETINE
	PHENELZINE SULFATE
	PROTRIPTYLINE
	SELEGILINE
	SERTRALINE
	TRANYLCYPROMINE
	TRIMIPRAMINE
	TRAZODONE
	VENLAFAXINE
	VILAZODONE
	VORTIOXETINE

© 2022 Leung LB et al. *JAMA Network Open.*

eTable 3. *Current Procedural Terminology (CPT)* Codes for Psychotherapy

CPT codes	*CPT* Name
90791	PSYCH DIAGNOSTIC EVALUATION
90792	PSYCH DIAG EVAL W/MED SRVCS
90832	PSYTX W PT 30 MINUTES
90833	PSYTX W PT W E/M 30 MIN
90834	PSYTX W PT 45 MINUTES
90836	PSYTX W PT W E/M 45 MIN
90837	PSYTX W PT 60 MINUTES
90838	PSYTX W PT W E/M 60 MIN
90839	PSYTX CRISIS INITIAL 60 MIN
90840	PSYTX CRISIS EA ADDL 30 MIN
90845	PSYCHOANALYSIS
90847	FAMILY PSYTX W/PT 50 MIN
90849	MULTIPLE FAMILY GROUP PSYTX
90853	GROUP PSYCHOTHERAPY
90857	INTAC GROUP PSYTX
90870	ELECTROCONVULSIVE THERAPY
90871	ELECTROCONVULSIVE THERAPY, MULTIPLE SEIZURES PER DAY
90875	PSYCHOPHYSIOLOGICAL THERAPY, 30 MIN
90876	PSYCHOPHYSIOLOGICAL THERAPY, 45 MIN

© 2022 Leung LB et al. *JAMA Network Open.*

eTable 4. VA Clinic Stop Codes for Mental Health Specialty Visits

Clinic stop code	MHS - Mental Health Service-related clinic stop codes description
165	BEREAVEMENT COUNSELING
292	OBSERVATION PSYCHIATRY
501	HOMELESS MENTALLY ILL OUTREACH
502	MENTAL HEALTH CLINIC- INDIVIDUAL
503	MENTAL HEALTH RESIDENTIAL CARE- INDIVIDUAL
504	GRANT AND DIEM - GROUP
505	DAY TREATMENT- INDIVIDUAL
506	DAY HOSPITAL- INDIVIDUAL
507	HUD/VASH- GROUP
508	HCHV/HCMI- GROUP
509	PSYCHIATRY
510	PSYCHOLOGY
511	GRANT AND PER DIEM- INDIVIDUAL
512	MENTAL HEALTH CONSULTATION
513	SUBSTANCE USE DISORDER- INDIVIDUAL
514	SUBSTANCE USE DISORDER- HOME VISIT
515	CWT/TR-HCMI
516	POST TRAUMATIC STRESS DISORDER (PTSD)– GROUP
517	CWT/SUBSTANCE ABUSE
518	CWT/TR-SUBSTANCE ABUSE
519	SUBSTANCE USE DISORDER/PTSD TEAMS
520	LONG-TERM ENHANCEMENT-INDIVIDUAL
521	LONG-TERM ENHANCEMENT-GROUP
522	DEPARTMENT OF HOUSING AND URBAN DEVELOPMENT (HUD)-VA SUPPORTED HOUSING (VASH) INDIVIDUAL
523	OPIOID TREATMENT PROGRAM
524	ACTIVE DUTY SEXUAL TRAUMA
525	WOMEN'S STRESS DISORDER TREATMENT TEAMS
526	TELEPHONE/SPECIAL PSYCHIATRY
527	TELEPHONE MH
528	TELEPHONE HOMELESS CHRONICALLY MENTALLY ILL (HCMI)
529	HEALTH CARE FOR HOMELESS VETERANS (HCHV)/HCMI INDIVIDUAL
530	TELEPHONE HUD-VASH
531	PRI CARE FOR PTS WITH SMI
532	PSYCHOSOCIAL REHABILITATION- INDIVIDUAL
533	MH INTERVENTION BIOMEDICAL CARE- INDIVIDUAL
534	MENTAL HEALTH INTEGRATED CARE- INDIVIDUAL
535	MENTAL HEALTH VOCATIONAL ASSISTANCE - INDIVIDUAL
536	TELEPHONE MH VOCATIONAL ASSISTANCE

537	TELEPHONE PSYCHOSOCIAL REHABILITATION
538	PSYCHOLOGICAL TESTING
539	MH INTEGRATED CARE- GROUP
540	PTSD CLINICAL TEAM (PCT) POST– TRAUMATIC STRESS INDVIDUAL
541	PTSD POST-TRAUMATIC STRESS
542	TELEPHONE PTSD
543	TELEPHONE/ALCOHOL DEPENDENCE
544	TELEPHONE/DRUG DEPENDENCE
545	TELEPHONE/SUBSTANCE USE DISORDER
546	TELEPHONE MH INTENSIVE CARE MANAGEMENT (MHICM)
547	INTENSIVE SUBSTANCE USE DISORDER- GROUP
548	INTENSIVE SUBSTANCE USE DISORDER- INDIVIDUAL
550	MENTAL HEALTH CLINIC- GROUP
551	IPCC COMMUNITY CLINIC/ DAY PROGRAM VISIT
552	MENTAL HEALTH INTENSIVE CASE MANAGEMENT (MHICM)- INDIVIDUAL
553	DAY TREATMENT- GROUP
554	DAY HOSPITAL- GROUP
555	HOMELESS VT COM EMP SVC INDIV
556	HOMELESS VT COM EMP SVC GRP
557	PSYCHIATRY- GROUP
558	PSYCHOLOGY- GROUP
559	PSYCHOSOCIAL REHABILITATION- GROUP
560	SUBSTANCE USE DISORDER- GROUP
561	PCT- POST TRAUMATIC STRESS- GROUP
562	PTSD- INDIVIDUAL
563	MENTAL HEALTH PRIMARY CARE - GROUP
564	MENTAL HEALTH TEAM CASE MANAGEMENT
565	MH INTERVENTION BIOMEDICAL CARE- GROUP
566	MENTAL HEALTH RISK-FACTOR REDUCTION EDUCATIONAL GROUP
567	MENTAL HEALTH INTENSIVE CASE MANAGEMENT (MHICM) GROUP
568	MENTAL HEALTH COMPENSATED WORK THERAPY/ SUPPORTED EMPLOYMENT (CWT/SE) FACE-TO-FACE
569	MENTAL HEALTH COMPENSATED WORK THERAPY/SUPPORTED EMPLOYMENT (CWT/SE) NON FACE-TO-FACE CBO NON-COUNT
570	MENTAL HEALTH COMPENSATED WORK THERAPY/ TRANSITIONAL WORK EXPERIENCE (CWT/TWE) NON FACE-TO-FACE CBO NON-COUNT
571	SERV-MH (SERVICES FOR RETURNING VETERANS-MENTAL HEALTH)- INDIVIDUAL
572	SERV-MH (SERVICES FOR RETURNING VETERANS-MENTAL HEALTH)- GROUP
573	MENTAL HEALTH INCENTIVE THERAPY FACE-TO-FACE
574	MENTAL HEALTH COMPENSATED WORK THERAPY/TRANSITIONAL WORK EXPERIENCE (CWT/TWE) FACE-TO-FACE
575	MENTAL HEALTH VOCATIONAL ASSISTANCE- GROUP
576	PSYCHOGERIATRIC CLINIC- INDIVIDUAL

© 2022 Leung LB et al. *JAMA Network Open.*

577	PSYCHOGERIATRIC CLINIC- GROUP
578	PSYCHOGERIATRIC DAY PROGRAM
579	TELEPHONE PSYCHOGERIATRICS
580	PTSD DAY HOSPITAL
581	PTSD DAY TREATMENT
582	PSYCHOSOCIAL REHABILITATION RECOVERY CENTER (PRRC)- INDIVIDUAL
583	PSYCHOSOCIAL REHABILITATION RECOVERY CENTER (PRRC)- GROUP
584	TELEPHONE PSYCHOSOCIAL REHABILITATION RECOVERY CENTER (PRRC)
586	RESIDENTIAL REHABILITATION TREATMENT PROGRAM (RRTP) - INDIVIDUAL
587	RESIDENTIAL REHABILITATION TREATMENT PROGRAM (RRTP) - GROUP
588	RESIDENTIAL REHABILITATION TREATMENT PROGRAM (RRTP) AFTERCARE-INDIVIDUAL
589	NON-ACTIVE DUTY SEXUAL TRAUMA
590	COMMUNITY OUTREACH HOMELESS VETS BY STAFF OTHER THAN HCHV AND RRTP PROGRAMS
591	INCARCERATED VETERANS RE-ENTRY
592	VETERANS JUSTICE OUTREACH
593	RESIDENTIAL REHABILITATION TREATMENT PROGRAM (RRTP) OUTREACH SERVICES
594	RESIDENTIAL REHABILITATION TREATMENT PROGRAM (RRTP) AFTERCARE - COMMUNITY
595	RESIDENTIAL REHABILITATION TREATMENT PROGRAM (RRTP) AFTERCARE-GROUP
596	RESIDENTIAL REHABILITATION TREATMENT PROGRAM (RRTP) ADMISSION SCREENING SERVICES
597	TELEPHONE/RESIDENTIAL REHABILITATION TREATMENT PROGRAM (RRTP)
598	RESIDENTIAL REHABILITATION TREATMENT PROGRAM (RRTP) OUTPATIENT - INDIVIDUAL
599	RESIDENTIAL REHABILITATION TREATMENT PROGRAM (RRTP) OUTPATIENT - GROUP

© 2022 Leung LB et al. *JAMA Network Open.*

eTable 5. *ICD-10* Codes Used for Other Mental Health Diagnoses in Study

PTSD_VA	PTSD_VA_Desc
F43.10	POST-TRAUMATIC STRESS DISORDER, UNSPECIFIED
F43.11	POST-TRAUMATIC STRESS DISORDER, ACUTE
F43.12	POST-TRAUMATIC STRESS DISORDER, CHRONIC
ETOH_VA	ETOH_VA_Desc
F10.10	ALCOHOL ABUSE, UNCOMPLICATED
F10.120	ALCOHOL ABUSE WITH INTOXICATION, UNCOMPLICATED
F10.121	ALCOHOL ABUSE WITH INTOXICATION DELIRIUM
F10.129	ALCOHOL ABUSE WITH INTOXICATION, UNSPECIFIED
F10.14	ALCOHOL ABUSE WITH ALCOHOL-INDUCED MOOD DISORDER
F10.150	ALCOHOL ABUSE WITH ALCOHOL-INDUCED PSYCHOTIC DISORDER WITH DELUSIONS
F10.151	ALCOHOL ABUSE WITH ALCOHOL-INDUCED PSYCHOTIC DISORDER WITH HALLUCINATIONS
F10.159	ALCOHOL ABUSE WITH ALCOHOL-INDUCED PSYCHOTIC DISORDER, UNSPECIFIED
F10.180	ALCOHOL ABUSE WITH ALCOHOL-INDUCED ANXIETY DISORDER
F10.181	ALCOHOL ABUSE WITH ALCOHOL-INDUCED SEXUAL DYSFUNCTION
F10.182	ALCOHOL ABUSE WITH ALCOHOL-INDUCED SLEEP DISORDER
F10.188	ALCOHOL ABUSE WITH OTHER ALCOHOL-INDUCED DISORDER
F10.19	ALCOHOL ABUSE WITH UNSPECIFIED ALCOHOL-INDUCED DISORDER
F10.20	ALCOHOL DEPENDENCE, UNCOMPLICATED
F10.21	ALCOHOL DEPENDENCE, IN REMISSION
F10.220	ALCOHOL DEPENDENCE WITH INTOXICATION, UNCOMPLICATED
F10.221	ALCOHOL DEPENDENCE WITH INTOXICATION DELIRIUM
F10.229	ALCOHOL DEPENDENCE WITH INTOXICATION, UNSPECIFIED
F10.230	ALCOHOL DEPENDENCE WITH WITHDRAWAL, UNCOMPLICATED
F10.231	ALCOHOL DEPENDENCE WITH WITHDRAWAL DELIRIUM
F10.232	ALCOHOL DEPENDENCE WITH WITHDRAWAL WITH PERCEPTUAL DISTURBANCE
F10.239	ALCOHOL DEPENDENCE WITH WITHDRAWAL, UNSPECIFIED
F10.24	ALCOHOL DEPENDENCE WITH ALCOHOL-INDUCED MOOD DISORDER
F10.250	ALCOHOL DEPENDENCE WITH ALCOHOL-INDUCED PSYCHOTIC DISORDER WITH DELUSIONS
F10.251	ALCOHOL DEPENDENCE WITH ALCOHOL-INDUCED PSYCHOTIC DISORDER WITH HALLUCINATIONS
F10.259	ALCOHOL DEPENDENCE WITH ALCOHOL-INDUCED PSYCHOTIC DISORDER, UNSPECIFIED
F10.26	ALCOHOL DEPENDENCE WITH ALCOHOL-INDUCED PERSISTING AMNESTIC DISORDER
F10.27	ALCOHOL DEPENDENCE WITH ALCOHOL-INDUCED PERSISTING DEMENTIA
F10.280	ALCOHOL DEPENDENCE WITH ALCOHOL-INDUCED ANXIETY DISORDER
F10.281	ALCOHOL DEPENDENCE WITH ALCOHOL-INDUCED SEXUAL DYSFUNCTION
F10.282	ALCOHOL DEPENDENCE WITH ALCOHOL-INDUCED SLEEP DISORDER

© 2022 Leung LB et al. *JAMA Network Open.*

F10.288	ALCOHOL DEPENDENCE WITH OTHER ALCOHOL-INDUCED DISORDER
F10.29	ALCOHOL DEPENDENCE WITH UNSPECIFIED ALCOHOL-INDUCED DISORDER
F10.920	ALCOHOL USE, UNSPECIFIED WITH INTOXICATION, UNCOMPLICATED
F10.921	ALCOHOL USE, UNSPECIFIED WITH INTOXICATION DELIRIUM
F10.929	ALCOHOL USE, UNSPECIFIED WITH INTOXICATION, UNSPECIFIED
F10.94	ALCOHOL USE, UNSPECIFIED WITH ALCOHOL-INDUCED MOOD DISORDER
F10.950	ALCOHOL USE, UNSPECIFIED WITH ALCOHOL-INDUCED PSYCHOTIC DISORDER WITH DELUSIONS
F10.951	ALCOHOL USE, UNSPECIFIED WITH ALCOHOL-INDUCED PSYCHOTIC DISORDER WITH HALLUCINATIONS
F10.959	ALCOHOL USE, UNSPECIFIED WITH ALCOHOL-INDUCED PSYCHOTIC DISORDER, UNSPECIFIED
F10.96	ALCOHOL USE, UNSPECIFIED WITH ALCOHOL-INDUCED PERSISTING AMNESTIC DISORDER
F10.97	ALCOHOL USE, UNSPECIFIED WITH ALCOHOL-INDUCED PERSISTING DEMENTIA
F10.980	ALCOHOL USE, UNSPECIFIED WITH ALCOHOL-INDUCED ANXIETY DISORDER
F10.981	ALCOHOL USE, UNSPECIFIED WITH ALCOHOL-INDUCED SEXUAL DYSFUNCTION
F10.982	ALCOHOL USE, UNSPECIFIED WITH ALCOHOL-INDUCED SLEEP DISORDER
F10.988	ALCOHOL USE, UNSPECIFIED WITH OTHER ALCOHOL-INDUCED DISORDER
F10.99	ALCOHOL USE, UNSPECIFIED WITH UNSPECIFIED ALCOHOL-INDUCED DISORDER
OPIA_VA	OPIA_VA_Desc
F11.10	OPIOID ABUSE, UNCOMPLICATED
F11.120	OPIOID ABUSE WITH INTOXICATION, UNCOMPLICATED
F11.121	OPIOID ABUSE WITH INTOXICATION DELIRIUM
F11.122	OPIOID ABUSE WITH INTOXICATION WITH PERCEPTUAL DISTURBANCE
F11.129	OPIOID ABUSE WITH INTOXICATION, UNSPECIFIED
F11.14	OPIOID ABUSE WITH OPIOID-INDUCED MOOD DISORDER
F11.150	OPIOID ABUSE WITH OPIOID-INDUCED PSYCHOTIC DISORDER WITH DELUSIONS
F11.151	OPIOID ABUSE WITH OPIOID-INDUCED PSYCHOTIC DISORDER WITH HALLUCINATIONS
F11.159	OPIOID ABUSE WITH OPIOID-INDUCED PSYCHOTIC DISORDER, UNSPECIFIED
F11.181	OPIOID ABUSE WITH OPIOID-INDUCED SEXUAL DYSFUNCTION
F11.182	OPIOID ABUSE WITH OPIOID-INDUCED SLEEP DISORDER
F11.188	OPIOID ABUSE WITH OTHER OPIOID-INDUCED DISORDER
F11.19	OPIOID ABUSE WITH UNSPECIFIED OPIOID-INDUCED DISORDER
F11.20	OPIOID DEPENDENCE, UNCOMPLICATED
F11.21	OPIOID DEPENDENCE, IN REMISSION
F11.220	OPIOID DEPENDENCE WITH INTOXICATION, UNCOMPLICATED
F11.221	OPIOID DEPENDENCE WITH INTOXICATION DELIRIUM
F11.222	OPIOID DEPENDENCE WITH INTOXICATION WITH PERCEPTUAL DISTURBANCE
F11.229	OPIOID DEPENDENCE WITH INTOXICATION, UNSPECIFIED
F11.23	OPIOID DEPENDENCE WITH WITHDRAWAL

© 2022 Leung LB et al. *JAMA Network Open.*

F11.24	OPIOID DEPENDENCE WITH OPIOID-INDUCED MOOD DISORDER
F11.250	OPIOID DEPENDENCE WITH OPIOID-INDUCED PSYCHOTIC DISORDER WITH DELUSIONS
F11.251	OPIOID DEPENDENCE WITH OPIOID-INDUCED PSYCHOTIC DISORDER WITH HALLUCINATIONS
F11.259	OPIOID DEPENDENCE WITH OPIOID-INDUCED PSYCHOTIC DISORDER, UNSPECIFIED
F11.281	OPIOID DEPENDENCE WITH OPIOID-INDUCED SEXUAL DYSFUNCTION
F11.282	OPIOID DEPENDENCE WITH OPIOID-INDUCED SLEEP DISORDER
F11.288	OPIOID DEPENDENCE WITH OTHER OPIOID-INDUCED DISORDER
F11.29	OPIOID DEPENDENCE WITH UNSPECIFIED OPIOID-INDUCED DISORDER
F11.90	OPIOID USE, UNSPECIFIED, UNCOMPLICATED
F11.920	OPIOID USE, UNSPECIFIED WITH INTOXICATION, UNCOMPLICATED
F11.921	OPIOID USE, UNSPECIFIED WITH INTOXICATION DELIRIUM
F11.922	OPIOID USE, UNSPECIFIED WITH INTOXICATION WITH PERCEPTUAL DISTURBANCE
F11.929	OPIOID USE, UNSPECIFIED WITH INTOXICATION, UNSPECIFIED
F11.93	OPIOID USE, UNSPECIFIED WITH WITHDRAWAL
F11.94	OPIOID USE, UNSPECIFIED WITH OPIOID-INDUCED MOOD DISORDER
F11.950	OPIOID USE, UNSPECIFIED WITH OPIOID-INDUCED PSYCHOTIC DISORDER WITH DELUSIONS
F11.951	OPIOID USE, UNSPECIFIED WITH OPIOID-INDUCED PSYCHOTIC DISORDER WITH HALLUCINATIONS
F11.959	OPIOID USE, UNSPECIFIED WITH OPIOID-INDUCED PSYCHOTIC DISORDER, UNSPECIFIED
F11.981	OPIOID USE, UNSPECIFIED WITH OPIOID-INDUCED SEXUAL DYSFUNCTION
F11.982	OPIOID USE, UNSPECIFIED WITH OPIOID-INDUCED SLEEP DISORDER
F11.988	OPIOID USE, UNSPECIFIED WITH OTHER OPIOID-INDUCED DISORDER
F11.99	OPIOID USE, UNSPECIFIED WITH UNSPECIFIED OPIOID-INDUCED DISORDER
CANN_VA	CANN_VA_Desc
F12.10	CANNABIS ABUSE, UNCOMPLICATED
F12.120	CANNABIS ABUSE WITH INTOXICATION, UNCOMPLICATED
F12.121	CANNABIS ABUSE WITH INTOXICATION DELIRIUM
F12.122	CANNABIS ABUSE WITH INTOXICATION WITH PERCEPTUAL DISTURBANCE
F12.129	CANNABIS ABUSE WITH INTOXICATION, UNSPECIFIED
F12.150	CANNABIS ABUSE WITH PSYCHOTIC DISORDER WITH DELUSIONS
F12.151	CANNABIS ABUSE WITH PSYCHOTIC DISORDER WITH HALLUCINATIONS
F12.159	CANNABIS ABUSE WITH PSYCHOTIC DISORDER, UNSPECIFIED
F12.180	CANNABIS ABUSE WITH CANNABIS-INDUCED ANXIETY DISORDER
F12.188	CANNABIS ABUSE WITH OTHER CANNABIS-INDUCED DISORDER
F12.19	CANNABIS ABUSE WITH UNSPECIFIED CANNABIS-INDUCED DISORDER
F12.20	CANNABIS DEPENDENCE, UNCOMPLICATED
F12.21	CANNABIS DEPENDENCE, IN REMISSION
F12.220	CANNABIS DEPENDENCE WITH INTOXICATION, UNCOMPLICATED
F12.221	CANNABIS DEPENDENCE WITH INTOXICATION DELIRIUM

© 2022 Leung LB et al. *JAMA Network Open.*

F12.222	CANNABIS DEPENDENCE WITH INTOXICATION WITH PERCEPTUAL DISTURBANCE
F12.229	CANNABIS DEPENDENCE WITH INTOXICATION, UNSPECIFIED
F12.250	CANNABIS DEPENDENCE WITH PSYCHOTIC DISORDER WITH DELUSIONS
F12.251	CANNABIS DEPENDENCE WITH PSYCHOTIC DISORDER WITH HALLUCINATIONS
F12.259	CANNABIS DEPENDENCE WITH PSYCHOTIC DISORDER, UNSPECIFIED
F12.280	CANNABIS DEPENDENCE WITH CANNABIS-INDUCED ANXIETY DISORDER
F12.288	CANNABIS DEPENDENCE WITH OTHER CANNABIS-INDUCED DISORDER
F12.29	CANNABIS DEPENDENCE WITH UNSPECIFIED CANNABIS-INDUCED DISORDER
F12.90	CANNABIS USE, UNSPECIFIED, UNCOMPLICATED
F12.920	CANNABIS USE, UNSPECIFIED WITH INTOXICATION, UNCOMPLICATED
F12.921	CANNABIS USE, UNSPECIFIED WITH INTOXICATION DELIRIUM
F12.922	CANNABIS USE, UNSPECIFIED WITH INTOXICATION WITH PERCEPTUAL DISTURBANCE
F12.929	CANNABIS USE, UNSPECIFIED WITH INTOXICATION, UNSPECIFIED
F12.950	CANNABIS USE, UNSPECIFIED WITH PSYCHOTIC DISORDER WITH DELUSIONS
F12.951	CANNABIS USE, UNSPECIFIED WITH PSYCHOTIC DISORDER WITH HALLUCINATIONS
F12.959	CANNABIS USE, UNSPECIFIED WITH PSYCHOTIC DISORDER, UNSPECIFIED
F12.980	CANNABIS USE, UNSPECIFIED WITH ANXIETY DISORDER
F12.988	CANNABIS USE, UNSPECIFIED WITH OTHER CANNABIS-INDUCED DISORDER
F12.99	CANNABIS USE, UNSPECIFIED WITH UNSPECIFIED CANNABIS-INDUCED DISORDER
SEDA_VA	SEDA_VA_Desc
F13.10	SEDATIVE, HYPNOTIC OR ANXIOLYTIC ABUSE, UNCOMPLICATED
F13.120	SEDATIVE, HYPNOTIC OR ANXIOLYTIC ABUSE WITH INTOXICATION, UNCOMPLICATED
F13.121	SEDATIVE, HYPNOTIC OR ANXIOLYTIC ABUSE WITH INTOXICATION DELIRIUM
F13.129	SEDATIVE, HYPNOTIC OR ANXIOLYTIC ABUSE WITH INTOXICATION, UNSPECIFIED
F13.14	SEDATIVE, HYPNOTIC OR ANXIOLYTIC ABUSE WITH SEDATIVE, HYPNOTIC OR ANXIOLYTIC-INDUCED MOOD DISORDER
F13.150	SEDATIVE, HYPNOTIC OR ANXIOLYTIC ABUSE WITH SEDATIVE, HYPNOTIC OR ANXIOLYTIC-INDUCED PSYCHOTIC DISORDER WITH DELUSIONS
F13.151	SEDATIVE, HYPNOTIC OR ANXIOLYTIC ABUSE WITH SEDATIVE, HYPNOTIC OR ANXIOLYTIC-INDUCED PSYCHOTIC DISORDER WITH HALLUCINATIONS
F13.159	SEDATIVE, HYPNOTIC OR ANXIOLYTIC ABUSE WITH SEDATIVE, HYPNOTIC OR ANXIOLYTIC-INDUCED PSYCHOTIC DISORDER, UNSPECIFIED
F13.180	SEDATIVE, HYPNOTIC OR ANXIOLYTIC ABUSE WITH SEDATIVE, HYPNOTIC OR ANXIOLYTIC-INDUCED ANXIETY DISORDER
F13.181	SEDATIVE, HYPNOTIC OR ANXIOLYTIC ABUSE WITH SEDATIVE, HYPNOTIC OR ANXIOLYTIC-INDUCED SEXUAL DYSFUNCTION
F13.182	SEDATIVE, HYPNOTIC OR ANXIOLYTIC ABUSE WITH SEDATIVE, HYPNOTIC OR ANXIOLYTIC-INDUCED SLEEP DISORDER
F13.188	SEDATIVE, HYPNOTIC OR ANXIOLYTIC ABUSE WITH OTHER SEDATIVE, HYPNOTIC OR ANXIOLYTIC-INDUCED DISORDER
F13.19	SEDATIVE, HYPNOTIC OR ANXIOLYTIC ABUSE WITH UNSPECIFIED SEDATIVE, HYPNOTIC OR ANXIOLYTIC-INDUCED DISORDER

© 2022 Leung LB et al. *JAMA Network Open.*

F13.20	SEDATIVE, HYPNOTIC OR ANXIOLYTIC DEPENDENCE, UNCOMPLICATED
F13.21	SEDATIVE, HYPNOTIC OR ANXIOLYTIC DEPENDENCE, IN REMISSION
F13.220	SEDATIVE, HYPNOTIC OR ANXIOLYTIC DEPENDENCE WITH INTOXICATION, UNCOMPLICATED
F13.221	SEDATIVE, HYPNOTIC OR ANXIOLYTIC DEPENDENCE WITH INTOXICATION DELIRIUM
F13.229	SEDATIVE, HYPNOTIC OR ANXIOLYTIC DEPENDENCE WITH INTOXICATION, UNSPECIFIED
F13.230	SEDATIVE, HYPNOTIC OR ANXIOLYTIC DEPENDENCE WITH WITHDRAWAL, UNCOMPLICATED
F13.231	SEDATIVE, HYPNOTIC OR ANXIOLYTIC DEPENDENCE WITH WITHDRAWAL DELIRIUM
F13.232	SEDATIVE, HYPNOTIC OR ANXIOLYTIC DEPENDENCE WITH WITHDRAWAL WITH PERCEPTUAL DISTURBANCE
F13.239	SEDATIVE, HYPNOTIC OR ANXIOLYTIC DEPENDENCE WITH WITHDRAWAL, UNSPECIFIED
F13.24	SEDATIVE, HYPNOTIC OR ANXIOLYTIC DEPENDENCE WITH SEDATIVE, HYPNOTIC OR ANXIOLYTIC-INDUCED MOOD DISORDER
F13.250	SEDATIVE, HYPNOTIC OR ANXIOLYTIC DEPENDENCE WITH SEDATIVE, HYPNOTIC OR ANXIOLYTIC-INDUCED PSYCHOTIC DISORDER WITH DELUSIONS
F13.251	SEDATIVE, HYPNOTIC OR ANXIOLYTIC DEPENDENCE WITH SEDATIVE, HYPNOTIC OR ANXIOLYTIC-INDUCED PSYCHOTIC DISORDER WITH HALLUCINATIONS
F13.259	SEDATIVE, HYPNOTIC OR ANXIOLYTIC DEPENDENCE WITH SEDATIVE, HYPNOTIC OR ANXIOLYTIC-INDUCED PSYCHOTIC DISORDER, UNSPECIFIED
F13.26	SEDATIVE, HYPNOTIC OR ANXIOLYTIC DEPENDENCE WITH SEDATIVE, HYPNOTIC OR ANXIOLYTIC-INDUCED PERSISTING AMNESTIC DISORDER
F13.27	SEDATIVE, HYPNOTIC OR ANXIOLYTIC DEPENDENCE WITH SEDATIVE, HYPNOTIC OR ANXIOLYTIC-INDUCED PERSISTING DEMENTIA
F13.280	SEDATIVE, HYPNOTIC OR ANXIOLYTIC DEPENDENCE WITH SEDATIVE, HYPNOTIC OR ANXIOLYTIC-INDUCED ANXIETY DISORDER
F13.281	SEDATIVE, HYPNOTIC OR ANXIOLYTIC DEPENDENCE WITH SEDATIVE, HYPNOTIC OR ANXIOLYTIC-INDUCED SEXUAL DYSFUNCTION
F13.282	SEDATIVE, HYPNOTIC OR ANXIOLYTIC DEPENDENCE WITH SEDATIVE, HYPNOTIC OR ANXIOLYTIC-INDUCED SLEEP DISORDER
F13.288	SEDATIVE, HYPNOTIC OR ANXIOLYTIC DEPENDENCE WITH OTHER SEDATIVE, HYPNOTIC OR ANXIOLYTIC-INDUCED DISORDER
F13.29	SEDATIVE, HYPNOTIC OR ANXIOLYTIC DEPENDENCE WITH UNSPECIFIED SEDATIVE, HYPNOTIC OR ANXIOLYTIC-INDUCED DISORDER
F13.90	SEDATIVE, HYPNOTIC, OR ANXIOLYTIC USE, UNSPECIFIED, UNCOMPLICATED
F13.920	SEDATIVE, HYPNOTIC OR ANXIOLYTIC USE, UNSPECIFIED WITH INTOXICATION, UNCOMPLICATED
F13.921	SEDATIVE, HYPNOTIC OR ANXIOLYTIC USE, UNSPECIFIED WITH INTOXICATION DELIRIUM
F13.929	SEDATIVE, HYPNOTIC OR ANXIOLYTIC USE, UNSPECIFIED WITH INTOXICATION, UNSPECIFIED
F13.930	SEDATIVE, HYPNOTIC OR ANXIOLYTIC USE, UNSPECIFIED WITH WITHDRAWAL, UNCOMPLICATED
F13.931	SEDATIVE, HYPNOTIC OR ANXIOLYTIC USE, UNSPECIFIED WITH WITHDRAWAL DELIRIUM
F13.932	SEDATIVE, HYPNOTIC OR ANXIOLYTIC USE, UNSPECIFIED WITH WITHDRAWAL WITH PERCEPTUAL DISTURBANCES

F13.939	SEDATIVE, HYPNOTIC OR ANXIOLYTIC USE, UNSPECIFIED WITH WITHDRAWAL, UNSPECIFIED
F13.94	SEDATIVE, HYPNOTIC OR ANXIOLYTIC USE, UNSPECIFIED WITH SEDATIVE, HYPNOTIC OR ANXIOLYTIC-INDUCED MOOD DISORDER
F13.950	SEDATIVE, HYPNOTIC OR ANXIOLYTIC USE, UNSPECIFIED WITH SEDATIVE, HYPNOTIC OR ANXIOLYTIC-INDUCED PSYCHOTIC DISORDER WITH DELUSIONS
F13.951	SEDATIVE, HYPNOTIC OR ANXIOLYTIC USE, UNSPECIFIED WITH SEDATIVE, HYPNOTIC OR ANXIOLYTIC-INDUCED PSYCHOTIC DISORDER WITH HALLUCINATIONS
F13.959	SEDATIVE, HYPNOTIC OR ANXIOLYTIC USE, UNSPECIFIED WITH SEDATIVE, HYPNOTIC OR ANXIOLYTIC-INDUCED PSYCHOTIC DISORDER, UNSPECIFIED
F13.96	SEDATIVE, HYPNOTIC OR ANXIOLYTIC USE, UNSPECIFIED WITH SEDATIVE, HYPNOTIC OR ANXIOLYTIC-INDUCED PERSISTING AMNESTIC DISORDER
F13.97	SEDATIVE, HYPNOTIC OR ANXIOLYTIC USE, UNSPECIFIED WITH SEDATIVE, HYPNOTIC OR ANXIOLYTIC-INDUCED PERSISTING DEMENTIA
F13.980	SEDATIVE, HYPNOTIC OR ANXIOLYTIC USE, UNSPECIFIED WITH SEDATIVE, HYPNOTIC OR ANXIOLYTIC-INDUCED ANXIETY DISORDER
F13.981	SEDATIVE, HYPNOTIC OR ANXIOLYTIC USE, UNSPECIFIED WITH SEDATIVE, HYPNOTIC OR ANXIOLYTIC-INDUCED SEXUAL DYSFUNCTION
F13.982	SEDATIVE, HYPNOTIC OR ANXIOLYTIC USE, UNSPECIFIED WITH SEDATIVE, HYPNOTIC OR ANXIOLYTIC-INDUCED SLEEP DISORDER
F13.988	SEDATIVE, HYPNOTIC OR ANXIOLYTIC USE, UNSPECIFIED WITH OTHER SEDATIVE, HYPNOTIC OR ANXIOLYTIC-INDUCED DISORDER
F13.99	SEDATIVE, HYPNOTIC OR ANXIOLYTIC USE, UNSPECIFIED WITH UNSPECIFIED SEDATIVE, HYPNOTIC OR ANXIOLYTIC-INDUCED DISORDER
COKE_VA	COKE_VA_Desc
F14.10	COCAINE ABUSE, UNCOMPLICATED
F14.120	COCAINE ABUSE WITH INTOXICATION, UNCOMPLICATED
F14.121	COCAINE ABUSE WITH INTOXICATION WITH DELIRIUM
F14.122	COCAINE ABUSE WITH INTOXICATION WITH PERCEPTUAL DISTURBANCE
F14.129	COCAINE ABUSE WITH INTOXICATION, UNSPECIFIED
F14.14	COCAINE ABUSE WITH COCAINE-INDUCED MOOD DISORDER
F14.150	COCAINE ABUSE WITH COCAINE-INDUCED PSYCHOTIC DISORDER WITH DELUSIONS
F14.151	COCAINE ABUSE WITH COCAINE-INDUCED PSYCHOTIC DISORDER WITH HALLUCINATIONS
F14.159	COCAINE ABUSE WITH COCAINE-INDUCED PSYCHOTIC DISORDER, UNSPECIFIED
F14.180	COCAINE ABUSE WITH COCAINE-INDUCED ANXIETY DISORDER
F14.181	COCAINE ABUSE WITH COCAINE-INDUCED SEXUAL DYSFUNCTION
F14.182	COCAINE ABUSE WITH COCAINE-INDUCED SLEEP DISORDER
F14.188	COCAINE ABUSE WITH OTHER COCAINE-INDUCED DISORDER
F14.19	COCAINE ABUSE WITH UNSPECIFIED COCAINE-INDUCED DISORDER
F14.20	COCAINE DEPENDENCE, UNCOMPLICATED
F14.21	COCAINE DEPENDENCE, IN REMISSION
F14.220	COCAINE DEPENDENCE WITH INTOXICATION, UNCOMPLICATED

F14.221	COCAINE DEPENDENCE WITH INTOXICATION DELIRIUM
F14.222	COCAINE DEPENDENCE WITH INTOXICATION WITH PERCEPTUAL DISTURBANCE
F14.229	COCAINE DEPENDENCE WITH INTOXICATION, UNSPECIFIED
F14.23	COCAINE DEPENDENCE WITH WITHDRAWAL
F14.24	COCAINE DEPENDENCE WITH COCAINE-INDUCED MOOD DISORDER
F14.250	COCAINE DEPENDENCE WITH COCAINE-INDUCED PSYCHOTIC DISORDER WITH DELUSIONS
F14.251	COCAINE DEPENDENCE WITH COCAINE-INDUCED PSYCHOTIC DISORDER WITH HALLUCINATIONS
F14.259	COCAINE DEPENDENCE WITH COCAINE-INDUCED PSYCHOTIC DISORDER, UNSPECIFIED
F14.280	COCAINE DEPENDENCE WITH COCAINE-INDUCED ANXIETY DISORDER
F14.281	COCAINE DEPENDENCE WITH COCAINE-INDUCED SEXUAL DYSFUNCTION
F14.282	COCAINE DEPENDENCE WITH COCAINE-INDUCED SLEEP DISORDER
F14.288	COCAINE DEPENDENCE WITH OTHER COCAINE-INDUCED DISORDER
F14.29	COCAINE DEPENDENCE WITH UNSPECIFIED COCAINE-INDUCED DISORDER
F14.90	COCAINE USE, UNSPECIFIED, UNCOMPLICATED
F14.920	COCAINE USE, UNSPECIFIED WITH INTOXICATION, UNCOMPLICATED
F14.921	COCAINE USE, UNSPECIFIED WITH INTOXICATION DELIRIUM
F14.922	COCAINE USE, UNSPECIFIED WITH INTOXICATION WITH PERCEPTUAL DISTURBANCE
F14.929	COCAINE USE, UNSPECIFIED WITH INTOXICATION, UNSPECIFIED
F14.94	COCAINE USE, UNSPECIFIED WITH COCAINE-INDUCED MOOD DISORDER
F14.950	COCAINE USE, UNSPECIFIED WITH COCAINE-INDUCED PSYCHOTIC DISORDER WITH DELUSIONS
F14.951	COCAINE USE, UNSPECIFIED WITH COCAINE-INDUCED PSYCHOTIC DISORDER WITH HALLUCINATIONS
F14.959	COCAINE USE, UNSPECIFIED WITH COCAINE-INDUCED PSYCHOTIC DISORDER, UNSPECIFIED
F14.980	COCAINE USE, UNSPECIFIED WITH COCAINE-INDUCED ANXIETY DISORDER
F14.981	COCAINE USE, UNSPECIFIED WITH COCAINE-INDUCED SEXUAL DYSFUNCTION
F14.982	COCAINE USE, UNSPECIFIED WITH COCAINE-INDUCED SLEEP DISORDER
F14.988	COCAINE USE, UNSPECIFIED WITH OTHER COCAINE-INDUCED DISORDER
F14.99	COCAINE USE, UNSPECIFIED WITH UNSPECIFIED COCAINE-INDUCED DISORDER
AMPH_VA	AMPH_VA_Desc
F15.10	OTHER STIMULANT ABUSE, UNCOMPLICATED
F15.20	OTHER STIMULANT DEPENDENCE, UNCOMPLICATED
F15.929	OTHER STIMULANT USE, UNSPECIFIED WITH INTOXICATION, UNSPECIFIED
HALL_VA	HALL_VA_Desc
F16.10	HALLUCINOGEN ABUSE, UNCOMPLICATED
F16.20	HALLUCINOGEN DEPENDENCE, UNCOMPLICATED

© 2022 Leung LB et al. *JAMA Network Open.*

NICO_VA	NICO_VA_Desc
F17.200	NICOTINE DEPENDENCE, UNSPECIFIED, UNCOMPLICATED
OTHD_VA	OTHD_VA_Desc
F18.10	INHALANT ABUSE, UNCOMPLICATED
F18.20	INHALANT DEPENDENCE, UNCOMPLICATED
F19.10	OTHER PSYCHOACTIVE SUBSTANCE ABUSE, UNCOMPLICATED
F19.120	OTHER PSYCHOACTIVE SUBSTANCE ABUSE WITH INTOXICATION, UNCOMPLICATED
F19.121	OTHER PSYCHOACTIVE SUBSTANCE ABUSE WITH INTOXICATION DELIRIUM
F19.122	OTHER PSYCHOACTIVE SUBSTANCE ABUSE WITH INTOXICATION WITH PERCEPTUAL DISTURBANCES
F19.129	OTHER PSYCHOACTIVE SUBSTANCE ABUSE WITH INTOXICATION, UNSPECIFIED
F19.14	OTHER PSYCHOACTIVE SUBSTANCE ABUSE WITH PSYCHOACTIVE SUBSTANCE-INDUCED MOOD DISORDER
F19.150	OTHER PSYCHOACTIVE SUBSTANCE ABUSE WITH PSYCHOACTIVE SUBSTANCE-INDUCED PSYCHOTIC DISORDER WITH DELUSIONS
F19.151	OTHER PSYCHOACTIVE SUBSTANCE ABUSE WITH PSYCHOACTIVE SUBSTANCE-INDUCED PSYCHOTIC DISORDER WITH HALLUCINATIONS
F19.159	OTHER PSYCHOACTIVE SUBSTANCE ABUSE WITH PSYCHOACTIVE SUBSTANCE-INDUCED PSYCHOTIC DISORDER, UNSPECIFIED
F19.16	OTHER PSYCHOACTIVE SUBSTANCE ABUSE WITH PSYCHOACTIVE SUBSTANCE-INDUCED PERSISTING AMNESTIC DISORDER
F19.17	OTHER PSYCHOACTIVE SUBSTANCE ABUSE WITH PSYCHOACTIVE SUBSTANCE-INDUCED PERSISTING DEMENTIA
F19.180	OTHER PSYCHOACTIVE SUBSTANCE ABUSE WITH PSYCHOACTIVE SUBSTANCE-INDUCED ANXIETY DISORDER
F19.181	OTHER PSYCHOACTIVE SUBSTANCE ABUSE WITH PSYCHOACTIVE SUBSTANCE-INDUCED SEXUAL DYSFUNCTION
F19.182	OTHER PSYCHOACTIVE SUBSTANCE ABUSE WITH PSYCHOACTIVE SUBSTANCE-INDUCED SLEEP DISORDER
F19.188	OTHER PSYCHOACTIVE SUBSTANCE ABUSE WITH OTHER PSYCHOACTIVE SUBSTANCE-INDUCED DISORDER
F19.19	OTHER PSYCHOACTIVE SUBSTANCE ABUSE WITH UNSPECIFIED PSYCHOACTIVE SUBSTANCE-INDUCED DISORDER
F19.20	OTHER PSYCHOACTIVE SUBSTANCE DEPENDENCE, UNCOMPLICATED
F19.21	OTHER PSYCHOACTIVE SUBSTANCE DEPENDENCE, IN REMISSION
F19.220	OTHER PSYCHOACTIVE SUBSTANCE DEPENDENCE WITH INTOXICATION, UNCOMPLICATED
F19.221	OTHER PSYCHOACTIVE SUBSTANCE DEPENDENCE WITH INTOXICATION DELIRIUM
F19.222	OTHER PSYCHOACTIVE SUBSTANCE DEPENDENCE WITH INTOXICATION WITH PERCEPTUAL DISTURBANCE
F19.229	OTHER PSYCHOACTIVE SUBSTANCE DEPENDENCE WITH INTOXICATION, UNSPECIFIED
F19.230	OTHER PSYCHOACTIVE SUBSTANCE DEPENDENCE WITH WITHDRAWAL, UNCOMPLICATED
F19.231	OTHER PSYCHOACTIVE SUBSTANCE DEPENDENCE WITH WITHDRAWAL DELIRIUM

© 2022 Leung LB et al. *JAMA Network Open.*

F19.232	OTHER PSYCHOACTIVE SUBSTANCE DEPENDENCE WITH WITHDRAWAL WITH PERCEPTUAL DISTURBANCE
F19.239	OTHER PSYCHOACTIVE SUBSTANCE DEPENDENCE WITH WITHDRAWAL, UNSPECIFIED
F19.24	OTHER PSYCHOACTIVE SUBSTANCE DEPENDENCE WITH PSYCHOACTIVE SUBSTANCE-INDUCED MOOD DISORDER
F19.250	OTHER PSYCHOACTIVE SUBSTANCE DEPENDENCE WITH PSYCHOACTIVE SUBSTANCE-INDUCED PSYCHOTIC DISORDER WITH DELUSIONS
F19.251	OTHER PSYCHOACTIVE SUBSTANCE DEPENDENCE WITH PSYCHOACTIVE SUBSTANCE-INDUCED PSYCHOTIC DISORDER WITH HALLUCINATIONS
F19.259	OTHER PSYCHOACTIVE SUBSTANCE DEPENDENCE WITH PSYCHOACTIVE SUBSTANCE-INDUCED PSYCHOTIC DISORDER, UNSPECIFIED
F19.26	OTHER PSYCHOACTIVE SUBSTANCE DEPENDENCE WITH PSYCHOACTIVE SUBSTANCE-INDUCED PERSISTING AMNESTIC DISORDER
F19.27	OTHER PSYCHOACTIVE SUBSTANCE DEPENDENCE WITH PSYCHOACTIVE SUBSTANCE-INDUCED PERSISTING DEMENTIA
F19.280	OTHER PSYCHOACTIVE SUBSTANCE DEPENDENCE WITH PSYCHOACTIVE SUBSTANCE-INDUCED ANXIETY DISORDER
F19.281	OTHER PSYCHOACTIVE SUBSTANCE DEPENDENCE WITH PSYCHOACTIVE SUBSTANCE-INDUCED SEXUAL DYSFUNCTION
F19.282	OTHER PSYCHOACTIVE SUBSTANCE DEPENDENCE WITH PSYCHOACTIVE SUBSTANCE-INDUCED SLEEP DISORDER
F19.288	OTHER PSYCHOACTIVE SUBSTANCE DEPENDENCE WITH OTHER PSYCHOACTIVE SUBSTANCE-INDUCED DISORDER
F19.29	OTHER PSYCHOACTIVE SUBSTANCE DEPENDENCE WITH UNSPECIFIED PSYCHOACTIVE SUBSTANCE-INDUCED DISORDER
F19.90	OTHER PSYCHOACTIVE SUBSTANCE USE, UNSPECIFIED, UNCOMPLICATED
F19.920	OTHER PSYCHOACTIVE SUBSTANCE USE, UNSPECIFIED WITH INTOXICATION, UNCOMPLICATED
F19.921	OTHER PSYCHOACTIVE SUBSTANCE USE, UNSPECIFIED WITH INTOXICATION WITH DELIRIUM
F19.922	OTHER PSYCHOACTIVE SUBSTANCE USE, UNSPECIFIED WITH INTOXICATION WITH PERCEPTUAL DISTURBANCE
F19.929	OTHER PSYCHOACTIVE SUBSTANCE USE, UNSPECIFIED WITH INTOXICATION, UNSPECIFIED
F19.930	OTHER PSYCHOACTIVE SUBSTANCE USE, UNSPECIFIED WITH WITHDRAWAL, UNCOMPLICATED
F19.931	OTHER PSYCHOACTIVE SUBSTANCE USE, UNSPECIFIED WITH WITHDRAWAL DELIRIUM
F19.932	OTHER PSYCHOACTIVE SUBSTANCE USE, UNSPECIFIED WITH WITHDRAWAL WITH PERCEPTUAL DISTURBANCE
F19.939	OTHER PSYCHOACTIVE SUBSTANCE USE, UNSPECIFIED WITH WITHDRAWAL, UNSPECIFIED
F19.94	OTHER PSYCHOACTIVE SUBSTANCE USE, UNSPECIFIED WITH PSYCHOACTIVE SUBSTANCE-INDUCED MOOD DISORDER
F19.950	OTHER PSYCHOACTIVE SUBSTANCE USE, UNSPECIFIED WITH PSYCHOACTIVE SUBSTANCE-INDUCED PSYCHOTIC DISORDER WITH DELUSIONS

© 2022 Leung LB et al. *JAMA Network Open.*

F19.951	OTHER PSYCHOACTIVE SUBSTANCE USE, UNSPECIFIED WITH PSYCHOACTIVE SUBSTANCE-INDUCED PSYCHOTIC DISORDER WITH HALLUCINATIONS
F19.959	OTHER PSYCHOACTIVE SUBSTANCE USE, UNSPECIFIED WITH PSYCHOACTIVE SUBSTANCE-INDUCED PSYCHOTIC DISORDER, UNSPECIFIED
F19.96	OTHER PSYCHOACTIVE SUBSTANCE USE, UNSPECIFIED WITH PSYCHOACTIVE SUBSTANCE-INDUCED PERSISTING AMNESTIC DISORDER
F19.97	OTHER PSYCHOACTIVE SUBSTANCE USE, UNSPECIFIED WITH PSYCHOACTIVE SUBSTANCE-INDUCED PERSISTING DEMENTIA
F19.980	OTHER PSYCHOACTIVE SUBSTANCE USE, UNSPECIFIED WITH PSYCHOACTIVE SUBSTANCE-INDUCED ANXIETY DISORDER
F19.981	OTHER PSYCHOACTIVE SUBSTANCE USE, UNSPECIFIED WITH PSYCHOACTIVE SUBSTANCE-INDUCED SEXUAL DYSFUNCTION
F19.982	OTHER PSYCHOACTIVE SUBSTANCE USE, UNSPECIFIED WITH PSYCHOACTIVE SUBSTANCE-INDUCED SLEEP DISORDER
F19.988	OTHER PSYCHOACTIVE SUBSTANCE USE, UNSPECIFIED WITH OTHER PSYCHOACTIVE SUBSTANCE-INDUCED DISORDER
F19.99	OTHER PSYCHOACTIVE SUBSTANCE USE, UNSPECIFIED WITH UNSPECIFIED PSYCHOACTIVE SUBSTANCE-INDUCED DISORDER
BPAD_VA	BPAD_VA_Desc
F31.0	BIPOLAR DISORDER, CURRENT EPISODE HYPOMANIC
F31.10	BIPOLAR DISORDER, CURRENT EPISODE MANIC WITHOUT PSYCHOTIC FEATURES, UNSPECIFIED
F31.11	BIPOLAR DISORDER, CURRENT EPISODE MANIC WITHOUT PSYCHOTIC FEATURES, MILD
F31.12	BIPOLAR DISORDER, CURRENT EPISODE MANIC WITHOUT PSYCHOTIC FEATURES, MODERATE
F31.13	BIPOLAR DISORDER, CURRENT EPISODE MANIC WITHOUT PSYCHOTIC FEATURES, SEVERE
F31.2	BIPOLAR DISORDER, CURRENT EPISODE MANIC SEVERE WITH PSYCHOTIC FEATURES
F31.30	BIPOLAR DISORDER, CURRENT EPISODE DEPRESSED, MILD OR MODERATE SEVERITY, UNSPECIFIED
F31.31	BIPOLAR DISORDER, CURRENT EPISODE DEPRESSED, MILD
F31.32	BIPOLAR DISORDER, CURRENT EPISODE DEPRESSED, MODERATE
F31.4	BIPOLAR DISORDER, CURRENT EPISODE DEPRESSED, SEVERE, WITHOUT PSYCHOTIC FEATURES
F31.5	BIPOLAR DISORDER, CURRENT EPISODE DEPRESSED, SEVERE, WITH PSYCHOTIC FEATURES
F31.60	BIPOLAR DISORDER, CURRENT EPISODE MIXED, UNSPECIFIED
F31.61	BIPOLAR DISORDER, CURRENT EPISODE MIXED, MILD
F31.62	BIPOLAR DISORDER, CURRENT EPISODE MIXED, MODERATE
F31.63	BIPOLAR DISORDER, CURRENT EPISODE MIXED, SEVERE, WITHOUT PSYCHOTIC FEATURES
F31.64	BIPOLAR DISORDER, CURRENT EPISODE MIXED, SEVERE, WITH PSYCHOTIC FEATURES
F31.70	BIPOLAR DISORDER, CURRENTLY IN REMISSION, MOST RECENT EPISODE UNSPECIFIED
F31.71	BIPOLAR DISORDER, IN PARTIAL REMISSION, MOST RECENT EPISODE HYPOMANIC

F31.72	BIPOLAR DISORDER, IN FULL REMISSION, MOST RECENT EPISODE HYPOMANIC
F31.73	BIPOLAR DISORDER, IN PARTIAL REMISSION, MOST RECENT EPISODE MANIC
F31.74	BIPOLAR DISORDER, IN FULL REMISSION, MOST RECENT EPISODE MANIC
F31.75	BIPOLAR DISORDER, IN PARTIAL REMISSION, MOST RECENT EPISODE DEPRESSED
F31.76	BIPOLAR DISORDER, IN FULL REMISSION, MOST RECENT EPISODE DEPRESSED
F31.77	BIPOLAR DISORDER, IN PARTIAL REMISSION, MOST RECENT EPISODE MIXED
F31.78	BIPOLAR DISORDER, IN FULL REMISSION, MOST RECENT EPISODE MIXED
F31.81	BIPOLAR II DISORDER
F31.89	OTHER BIPOLAR DISORDER
F31.9	BIPOLAR DISORDER, UNSPECIFIED
SCHZ_VA	SCHZ_VA_Desc
F20.0	PARANOID SCHIZOPHRENIA
F20.1	DISORGANIZED SCHIZOPHRENIA
F20.2	CATATONIC SCHIZOPHRENIA
F20.3	UNDIFFERENTIATED SCHIZOPHRENIA
F20.5	RESIDUAL SCHIZOPHRENIA
F20.81	SCHIZOPHRENIFORM DISORDER
F20.89	OTHER SCHIZOPHRENIA
F20.9	SCHIZOPHRENIA, UNSPECIFIED
F21.	SCHIZOTYPAL DISORDER
F25.0	SCHIZOAFFECTIVE DISORDER, BIPOLAR TYPE
F25.1	SCHIZOAFFECTIVE DISORDER, DEPRESSIVE TYPE
F25.8	OTHER SCHIZOAFFECTIVE DISORDERS
F25.9	SCHIZOAFFECTIVE DISORDER, UNSPECIFIED
ANXI_VA	ANXI_VA_Desc
F40.00	AGORAPHOBIA, UNSPECIFIED
F40.01	AGORAPHOBIA WITH PANIC DISORDER
F40.02	AGORAPHOBIA WITHOUT PANIC DISORDER
F40.10	SOCIAL PHOBIA, UNSPECIFIED
F40.11	SOCIAL PHOBIA, GENERALIZED
F40.210	ARACHNOPHOBIA
F40.218	OTHER ANIMAL TYPE PHOBIA
F40.220	FEAR OF THUNDERSTORMS
F40.228	OTHER NATURAL ENVIRONMENT TYPE PHOBIA
F40.230	FEAR OF BLOOD
F40.231	FEAR OF INJECTIONS AND TRANSFUSIONS
F40.232	FEAR OF OTHER MEDICAL CARE
F40.233	FEAR OF INJURY
F40.240	CLAUSTROPHOBIA

© 2022 Leung LB et al. *JAMA Network Open.*

F40.241	ACROPHOBIA
F40.242	FEAR OF BRIDGES
F40.243	FEAR OF FLYING
F40.248	OTHER SITUATIONAL TYPE PHOBIA
F40.290	ANDROPHOBIA
F40.291	GYNEPHOBIA
F40.298	OTHER SPECIFIED PHOBIA
F40.8	OTHER PHOBIC ANXIETY DISORDERS
F40.9	PHOBIC ANXIETY DISORDER, UNSPECIFIED
F41.0	PANIC DISORDER [EPISODIC PAROXYSMAL ANXIETY] WITHOUT AGORAPHOBIA
F41.1	GENERALIZED ANXIETY DISORDER
F41.3	OTHER MIXED ANXIETY DISORDERS
F41.8	OTHER SPECIFIED ANXIETY DISORDERS
F41.9	ANXIETY DISORDER, UNSPECIFIED
F42.	OBSESSIVE-COMPULSIVE DISORDER
F42.2	MIXED OBSESSIONAL THOUGHTS AND ACTS
F42.3	HOARDING DISORDER
F42.4	EXCORIATION (SKIN-PICKING) DISORDER
F42.8	OTHER OBSESSIVE-COMPULSIVE DISORDER
F42.9	OBSESSIVE-COMPULSIVE DISORDER, UNSPECIFIED
F43.0	ACUTE STRESS REACTION
F43.20	ADJUSTMENT DISORDER, UNSPECIFIED
F43.21	ADJUSTMENT DISORDER WITH DEPRESSED MOOD
F43.22	ADJUSTMENT DISORDER WITH ANXIETY
F43.23	ADJUSTMENT DISORDER WITH MIXED ANXIETY AND DEPRESSED MOOD
F43.24	ADJUSTMENT DISORDER WITH DISTURBANCE OF CONDUCT
F43.25	ADJUSTMENT DISORDER WITH MIXED DISTURBANCE OF EMOTIONS AND CONDUCT
F43.29	ADJUSTMENT DISORDER WITH OTHER SYMPTOMS
F43.8	OTHER REACTIONS TO SEVERE STRESS
F43.9	REACTION TO SEVERE STRESS, UNSPECIFIED
F44.0	DISSOCIATIVE AMNESIA
F44.1	DISSOCIATIVE FUGUE
F44.2	DISSOCIATIVE STUPOR
F44.4	CONVERSION DISORDER WITH MOTOR SYMPTOM OR DEFICIT
F44.5	CONVERSION DISORDER WITH SEIZURES OR CONVULSIONS
F44.6	CONVERSION DISORDER WITH SENSORY SYMPTOM OR DEFICIT
F44.7	CONVERSION DISORDER WITH MIXED SYMPTOM PRESENTATION
F44.81	DISSOCIATIVE IDENTITY DISORDER
F44.89	OTHER DISSOCIATIVE AND CONVERSION DISORDERS
F44.9	DISSOCIATIVE AND CONVERSION DISORDER, UNSPECIFIED
F45.0	SOMATIZATION DISORDER

© 2022 Leung LB et al. *JAMA Network Open.*

F45.1	UNDIFFERENTIATED SOMATOFORM DISORDER
F45.20	HYPOCHONDRIACAL DISORDER, UNSPECIFIED
F45.21	HYPOCHONDRIASIS
F45.22	BODY DYSMORPHIC DISORDER
F45.29	OTHER HYPOCHONDRIACAL DISORDERS
F45.41	PAIN DISORDER EXCLUSIVELY RELATED TO PSYCHOLOGICAL FACTORS
F45.42	PAIN DISORDER WITH RELATED PSYCHOLOGICAL FACTORS
F45.8	OTHER SOMATOFORM DISORDERS
F45.9	SOMATOFORM DISORDER, UNSPECIFIED
F48.1	DEPERSONALIZATION-DEREALIZATION SYNDROME
F48.2	PSEUDOBULBAR AFFECT
F48.8	OTHER SPECIFIED NONPSYCHOTIC MENTAL DISORDERS
F48.9	NONPSYCHOTIC MENTAL DISORDER, UNSPECIFIED

© 2022 Leung LB et al. *JAMA Network Open.*

eTable 6. Associations Between Receipt of Timely Depression Follow-up and Treatment and Various Patient Characteristics (Among All Screen-Positive Patients)

	Follow-up with 84 days			Follow-up with 180 days			Minimally appropriate treatment		
n = 37,063 person-years	**OR**	**95% CI**	**p-value**	**OR**	**95% CI**	**p-value**	**OR**	**95% CI**	**p-value**
Patient Characteristics									
**Age**	0.98	.978-.983	<.001	0.98	0.975-0.981	<.001	0.976	0.97-0.98	<.001
**Sex**									
Men (ref)	-	-	-	-	-	-	-	-	-
Female	1.08	0.95-1.23	0.60	1.05	0.93-1.18	0.43	1.30	1.16-1.46	<.001
**Race-Ethnicity**									
White (ref)	-	-	-	-	-	-	-	-	-
Black	1.32	1.17-1.48	<.001	1.40	1.26-1.56	<.001	1.30	1.17-1.44	<.001
Hispanic	1.03	0.93-1.15	0.53	1.04	0.95-1.14	0.40	0.99	0.91-1.09	0.90
Other	1.10	0.90-1.34	0.37	1.17	1.04-1.31	0.01	1.07	0.91-1.25	0.44
Unknown/Missing	1.17	1.02-1.34	0.02	1.17	1.04-1.31	0.01	1.05	0.94-1.17	0.37
**Marital Status**									
Married (ref)	-	-	-	-	-	-	-	-	-
Single/Previously Married	0.98	.897-1.06	0.60	0.98	0.91-1.06	0.67	1.07	0.99-1.14	0.07
**Means Test**									
Exempt (ref)	-	-	-	-	-	-	-	-	-
Non-Exempt	1.01	.842-1.20	0.94	1.12	0.96-1.31	0.14	1.09	0.94-1.26	0.26
Any Copay Required	0.94	.809-1.08	0.36	0.98	0.87-1.11	0.79	0.91	0.81-1.02	0.26
Missing	1.05	.934-1.18	0.40	1.09	0.98-1.21	0.79	1.04	0.95-1.15	0.38
**Service-Connected Disability**									
0% (ref)	-	-	-	-	-	-	-	-	-
1-50%	0.97	.735-1.28	0.84	0.99	0.78-1.26	0.93	0.87	0.70-1.09	0.22
51-100%	0.56	.422-.733	<.001	0.63	0.49-0.79	<.001	0.69	0.55-0.85	<.001
Missing	1.26	.969-1.63	0.09	1.31	1.04-1.64	0.02	1.01	0.82-1.24	0.92
**Charlson Comorbidity Index**									
0 (ref)	-	-	-	-	-	-	-	-	-
1	0.91	.817-1.01	0.08	1.02	0.93-1.12	0.65	1.06	0.98-1.16	0.15
2+	0.81	.720-.910	<.001	0.93	0.84-1.02	0.14	1.03	0.94-1.12	0.53
**Mental Health/Substance Use Disorder**									
Anxiety Disorder	4.90	4.22-5.69	<.001	5.71	4.98-6.53	<.001	5.34	4.76-5.98	<.001
PTSD	3.85	3.36-4.40	<.001	5.23	4.59-5.96	<.001	5.26	4.70-5.90	<.001
Serious Mental Illness	3.40	2.76-4.18	<.001	5.21	4.22-6.43	<.001	4.91	4.00-6.02	<.001
Alcohol Use Disorder	2.27	1.98-2.60	<.001	2.31	2.04-2.62	<.001	2.07	1.84-2.32	<.001
Substance Use Disorder	2.32	1.96-2.76	<.001	2.35	1.99-2.77	<.001	2.45	2.07-2.90	<.001

© 2022 Leung LB et al. *JAMA Network Open.*
